# Occurrence and Reasons for On-Farm Emergency Slaughter (OFES) in Northern Italian Cattle †

**DOI:** 10.3390/ani15152239

**Published:** 2025-07-30

**Authors:** Francesca Fusi, Camilla Allegri, Alessandra Gregori, Claudio Monaci, Sara Gabriele, Tiziano Bernardo, Valentina Lorenzi, Claudia Romeo, Federico Scali, Lucia Scuri, Giorgio Bontempi, Maria Nobile, Luigi Bertocchi, Giovanni Loris Alborali, Adriana Ianieri, Sergio Ghidini

**Affiliations:** 1 Istituto Zooprofilattico Sperimentale della Lombardia e dell’Emilia-Romagna, Via Antonio Bianchi 9, 25124 Brescia, Italy; francesca.fusi@izsler.it (F.F.); sara.gabriele@izsler.it (S.G.); tiziano.bernardo@izsler.it (T.B.); valentina.lorenzi@izsler.it (V.L.); claudiarosa.romeo@izsler.it (C.R.); federico.scali@izsler.it (F.S.); luigi.bertocchi@izsler.it (L.B.); giovanni.alborali@izsler.it (G.L.A.); 2 Department of Food and Drug, Parma University, Via del Taglio 10, 43126 Parma, Italy; lucia.scuri@unipr.it (L.S.); adriana.ianieri@unipr.it (A.I.); 3 Department of Veterinary Medicine and Animal Sciences, University of Milan, Via dell’Università 6, 26900 Lodi, Italy; maria.nobile1@unimi.it (M.N.); sergio.ghidini@unimi.it (S.G.); 4 Department of Veterinary and Food Safety of Animal Origin, Agenzia di Tutela della Salute (ATS) di Brescia, Viale Duca degli Abruzzi 15, 25124 Brescia, Italy; alessandra.gregori@ats-brescia.it (A.G.); claudio.monaci@ats-brescia.it (C.M.); 5 Department of Economics and Management, University of Brescia, Via S. Faustino, 74b, 25122 Brescia, Italy; giorgio.bontempi@unibs.it

**Keywords:** cattle mortality, ante-mortem inspection, locomotion disorders, animal welfare legislation

## Abstract

On-farm emergency slaughter (OFES) is employed when cattle are unfit for transport but still suitable for human consumption, thereby ensuring animal welfare and reducing food waste. This study analysed OFES patterns in Northern Italy, where a large cattle population is housed but information on the practice is rarely analysed. A total of 12,052 OFES cases from 2021 to 2023 were analysed. Most involved female cattle (94%) from dairy farms (79%). Locomotor disorders were the leading reason (70%), particularly trauma and fractures, followed by recumbency (13%) and calving-related issues (10%). Post-mortem findings showed limbs and joints as the most frequent condemnation sites (36%), often linked to trauma. A significant reduction in OFES cases occurred over time, mainly due to fewer recumbency and calving issues, likely reflecting stricter eligibility criteria introduced in 2022. Weekly variations, with peaks on Mondays and lows on Saturdays, suggest that logistical constraints may sometimes influence OFES promptness. These findings suggest that on-farm management and animal handling could be improved further to reduce welfare risks and carcass waste. Due to the lack of standardised data collection and regulatory harmonisation, a multi-country investigation could improve our understanding of this topic and inform best practice.

## Simple Summary

On-farm emergency slaughter (OFES) is important to maintain the welfare of cattle unfit for transport, while reducing food waste and financial losses to farmers. Although Italy is a large cattle producer, little is known about OFES in the country. This study examines how and why this practice is used by analysing more than 12,000 cases of OFES from 2021 to 2023. Most cases involved dairy cattle, especially females with severe locomotor problems. Other common reasons were cattle being unable to stand (recumbency) or problems related to calving. After OFES, parts of the carcass are sometimes unfit for human consumption, most commonly the limbs and joints, usually due to injury. The number of OFES cases has gone down over time, especially for cows with calving problems or recumbency, likely due to stricter rules introduced in 2022. OFES were more frequent right after the weekend, suggesting that scheduling issues may sometimes take priority over animal welfare. This study highlights the need for improved farm practices, more standardised OFES criteria and, where possible, mobile slaughter units.

## 1. Introduction

The management of end-of-career cattle pose significant animal welfare concerns. Cows may have reached the end of their productive life on a given farm because they are no longer productive, even though they are still fertile and healthy. The farmer may decide to remove the animals from the herd by selling them to another farm or a slaughterhouse, in a planned and rational process known as ‘voluntary culling’ [1,2]. Reasons for voluntary culling typically relate to production or economic factors, such as low milk yield, poor milk quality, age, low genetic merit, aggressive behaviour, overstocking or high feed costs [3]. Conversely, ‘involuntary culling’ is the result of health issues or injuries that compromise the animal’s welfare and require urgent intervention. These include accidents or physical injuries causing acute pain (e.g., a fractured limb or splits), dystocia or post-calving complications (e.g., milk fever, palsy, metabolic disorders), as well as chronic conditions such as lameness, arthritis, mastitis or infectious diseases [4]. Involuntary culling often results in unplanned herd exits, raising both ethical and economic concerns [2,3]. This type of culling includes both cattle that leave the farm for slaughter and cattle that die on the farm [1]. The latter case is also recorded as on-farm mortality, which encompasses unassisted death, euthanasia and on-farm emergency slaughter (OFES) [5,6,7,8].

On-farm emergency slaughter is a practice employed when livestock, such as ungulates, are unfit for transport to a slaughterhouse but remain suitable for human consumption. On a global scale, OFES is permitted in certain regions, including the EU, the UK, Norway and Canada [9,10]. In contrast, this practice is not permitted in Australia and New Zealand, where euthanasia is the only option. These differences reflect regional variations in legislation and ethical considerations [11]. Furthermore, in the US, OFES is not recognised within the regulatory framework as an acceptable method of producing meat for human consumption (21 U.S.C. §§ 603, 610; 9 C.F.R. §§ 309.3(e), 313). Although legal frameworks for OFES may differ internationally, they should generally adhere to stringent veterinary and food safety standards, balancing animal welfare and public health [12]. Within the EU, OFES are regulated by EU and national legislation, which only applies to otherwise healthy animals that have suffered an accident that precludes their transport for welfare reasons (Regulation (EC) No. 853/2004 [13] and Council Regulation (EC) No. 1/2005) [14]. In particular, the management of acutely traumatised animals requires careful consideration of Available online:several options, including euthanasia, therapeutic treatment, and OFES. The decision-making process in such cases includes an assessment of the prognosis and feasibility of therapy [15].

While euthanasia and therapeutic interventions may be possible, OFES offers a clear economic advantage by allowing the carcass to be salvaged for human consumption, thereby reducing food waste and financial losses [10]. However, this economic incentive may contribute to the complex and often controversial perception of OFES by stakeholders and consumers [16,17,18]. Among the former, veterinarians may face practical and clinical challenges during the ante-mortem examinations (e.g., recumbent cattle can be difficult to examine), and may struggle to interpret signs and symptoms in order to properly understand the animal’s health and welfare condition [19,20]. Furthermore, practical constraints, such as the limited availability of veterinarians, the reluctance of some slaughterhouse operators to accept OFES carcasses, and the associated high costs, have hindered the wider application of this practice in certain regions [17,21]. Even among EU countries, where a common regulatory framework exists, there are still considerable differences in the availability of suitable slaughterhouses, transport practices for injured animals, and specific slaughter procedures [9]. Studies on OFES have been carried out in a few countries, such as the Czech Republic [22,23], Ireland [24], Canada [10] and Norway [20], but studies is still scarce for Mediterranean countries, such as Italy, which represents the fifth largest state in the EU in terms of bovine population, housing on average almost six million cattle [25]. This limited information highlights the need for a better understanding of how OFES is applied in Southern Europe, where differences in climate, structures and production may influence such practices. This study aims to address this gap by investigating the reasons, prevalence and temporal patterns of OFES practices in Italy.

## 2. Materials and Methods

### 2.1. Legal Framework

**Figure 1:**
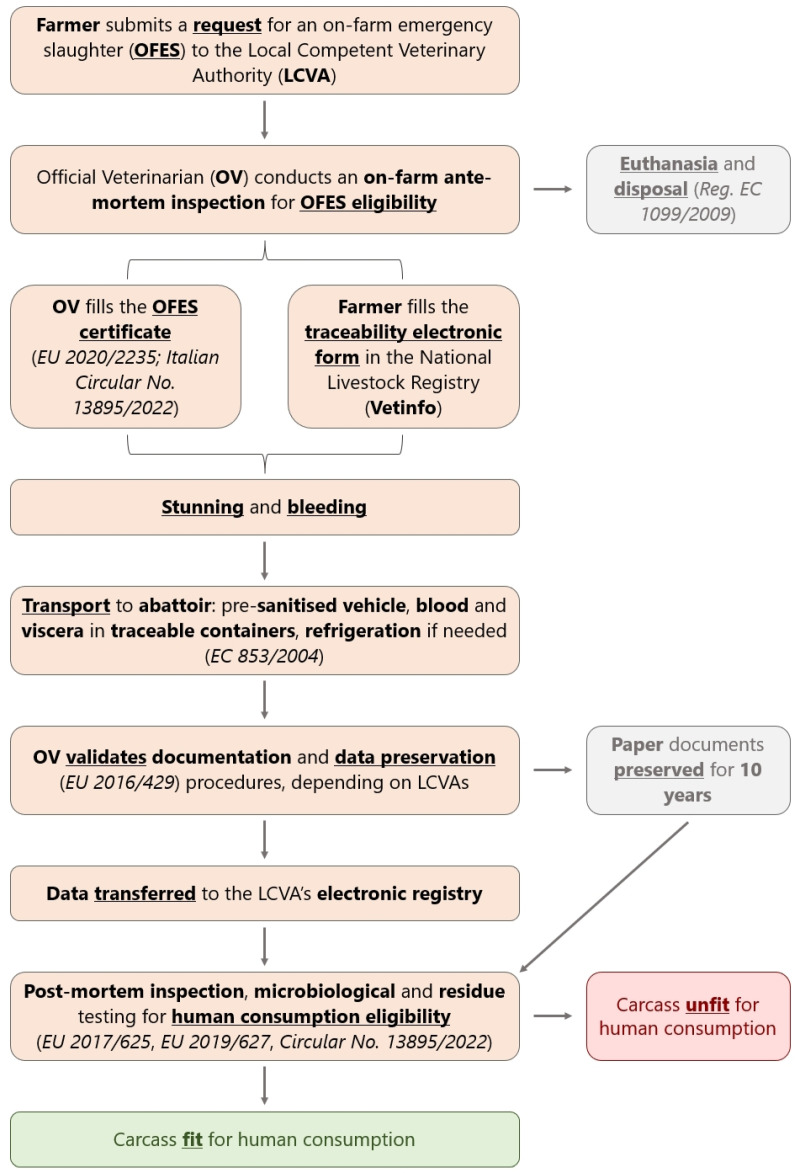
Flow chart summarising the decision-making process and the operations related to on-farm emergency slaughter (OFES) in Italy. The grey boxes show steps that were not pertinent to the study [30,31,32].

In Italy, OFES is regulated by a combination of EU (Regulations (EC) No. 853/2004 and No. 1099/2009 [26]; Commission Delegated Regulations (EU) 2019/624 [27] and 2020/2235 [28]) and national legislation (Italian Ministry of Health, Circular No. 13895/2022 [29]) (Table S1), which collectively establish requirements for ante-mortem and post-mortem inspections, the protection of farm animals and official certification procedures outside of the ordinary slaughterhouse settings. The procedural workflow is shown in Figure 1.

### 2.2. Data Collection and Management

The data for the study were provided by a LCVA in northern Italy (province of Brescia, Lombardy region), covering all OFES cases in cattle sent to slaughterhouses under this LCVA’s jurisdiction between 2021 and 2023. These data included the identification code of the farm and information on the slaughtered animal (ear tag number, date of birth and sex), the date of OFES and ante-mortem findings. Where available, post-mortem findings were also collected. Other farm information, such as the production system (dairy, beef or mixed) and the herd size, was retrieved from the National Livestock Register (Vetinfo) database. All data collected during the study were stored and managed using Microsoft Excel 365 (Microsoft Corporation, Redmond, WA, USA).

The age of each cattle was calculated by subtracting the date of birth from the date of slaughter and adding one day to account for the full lifespan. Female and male subjects were grouped as described in previous studies [20,33], age groups and corresponding age ranges are shown in Table 1.

Data on OFES reasons collected during ante-mortem inspections by the Official Veterinarians were manually categorised into four primary categories (‘locomotion’, ‘recumbency’, ‘calving-related problems’ and ‘other’) and thirty sub-categories (modified from [20,24]), as reported in Table 2. The latter were identified using clinical descriptions from veterinary certificates and consistent inclusion criteria for each reason [20].

Post-mortem findings were grouped into 11 primary categories according to the exclusion localisation (‘gastro-intestinal system and peritoneum’, ’head and oral cavity’, ‘heart and pericardium’, ‘integumentary system and mammary gland’, limbs and joints’, ‘liver and hepatic lymph nodes’, ‘respiratory system’, ‘spleen and kidneys’, ‘multiorgan’, ‘half or whole carcass’ and ‘empty or illogical’) and then combined with the reason why they were deemed unfit for human consumption, as shown in Table 3, to create several subcategories.

### 2.3. Statistical Analysis

The overall temporal trends in the number of OFES were analysed on the full dataset (*N* = 12,052) through a Poisson regression, including year, season and day of the week as explanatory variables. Pairwise contrasts of marginal means were carried out for each predictor, applying a Holm correction for multiple comparisons. We then tested the association of categories and subcategories with year, season, production type and age group by means of Pearson’s chi square tests. The strength of any significant association was estimated through Cramér’s V and standardised residuals were examined to estimate the contribution of each pair to the chi square statistic [34]. The analysis of OFES categories was limited to the four most prevalent age groups (i.e., heifer, young cow, old cow and bull calf, *N* = 11,727), and when analysing subcategories, this subset was further limited to the six subcategories with prevalence > 5% (*N* = 9374). Finally, by using the same approach we also tested the association of OFES categories with the outcome of the available post-mortem inspections. This analysis was limited to the subset of exclusion reasons and exclusion localisations with prevalence > 2% (*N* = 2766). All the analyses and visualisations were carried out using R Statistical Software (v4.5.1; R Core Team 2023, R Foundation for Statistical Computing, Vienna, Austria).

## 3. Results

During the period 2021–2023, a total of 12,052 carcasses subjected to OFES were sent to 20 slaughterhouses under the jurisdiction of the LCVA of the province of Brescia. The OFES were carried out in 1858 cattle farms located in Northern Italy. The median herd size was 255 cattle with an interquartile range of 297.

Overall, 11,359 (94.25%) OFES cases were performed on female cattle, and dairy production accounted for 9514 (78.94%) of all cases. Details of the OFES for each year according to production system, sex and animal category are reported in Table 4.

Among the 11,359 OFES cases performed on female cattle, 9467 (83.34%) occurred on dairy farms, 1396 (12.29%) on mixed farms, and 496 (4.37%) on beef farms. In contrast, out of the 693 OFES procedures carried out on male cattle, 545 (78.64%) took place in beef farms, 101 (14.57%) in mixed farms, and 47 (6.78%) in dairy farms. The detailed distributions of the OFES cases for each production system according to sex and animal category are provided in Table S2.

Out of the 12,052 OFES reasons recorded during the study, locomotion issues were the most common reasons, accounting for 8362 cases (69.37%); recumbency was observed in 1597 cases (13.24%) and calving-related problems were seen in 1252 cases (10.39%), while other reasons contributed to the remaining 841 cases (6.98%). Detailed reasons (categories and sub-categories) are reported in Table 5. Details on the body localisation of injuries within the locomotion category are reported in Table S3.

Information on post-mortem examinations was retrieved in 3181 out of 12,052 (26.39%) OFES cases carried out in 1061 farms. The most common condemnation anatomical sites, accounting for 71.33% of all exclusions, were limbs and joints (35.81%), liver (21.25%) and respiratory system (14.27%). The distribution of all sites is shown in Table 6.

The three most common reasons for condemnation, accounting for 74.98% of the total, were haematomas (34.58%), inflammatory disorders (23.48%) and circulatory-metabolic disorders (16.91%). The detailed distribution of condemnations for each localisation is reported in Table S4.

**Figure 2:**
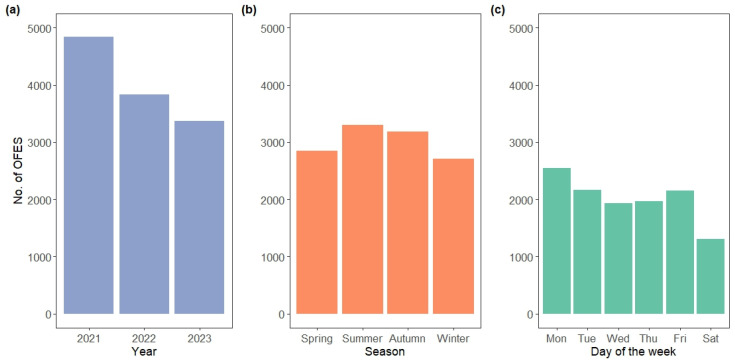
Histograms showing the distribution of 12,052 on-farm emergency slaughter (OFES) instances carried out on 1858 cattle farms in Northern Italy by (**a**) year; (**b**) season; and (**c**) day of the week.

The number of OFES decreased progressively during the study period (*p* < 0.0001; Figure 2a), with an estimated 33% (±2%) reduction from 2021 to 2023 (*p_ad_*_j_ < 0.0001). Significant differences in the occurrence of OFES across seasons and between the days of the week were also detected (both *p* < 0.0001). OFES were more frequent during the summer and autumn and less common in winter (winter vs. summer: −17% ± 2%, *p_adj_* < 0.0001) (Figure 2b). OFES also peaked on Mondays, were equally frequent during the middle days of the week, but then significantly decreased on Saturdays (Saturday vs. Monday: −43% ± 2%, *p_ad_*_j_ < 0.0001) (Figure 2c).

**Figure 3:**
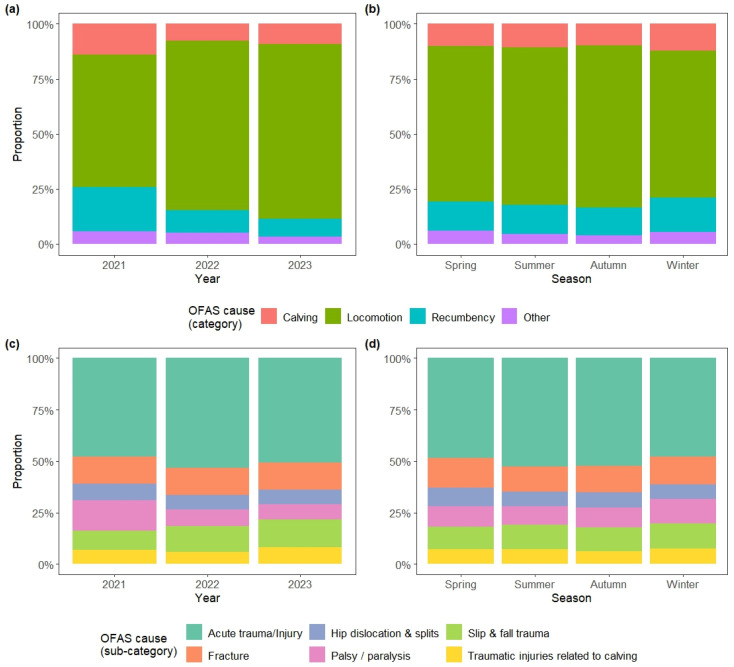
Temporal trends in the categories and sub-categories of reasons for on-farm emergency slaughter (OFES) instances: (**a**) categories by year; (**b**) categories by season; (**c**) subcategories by year; (**d**) subcategories by season.

Although the effect sizes were small, with V values ranging from 0.04 to 0.13, the categories and subcategories of OFES reasons were significantly associated with all the examined variables. In detail, the categories of OFES reasons varied significantly over the years (V = 0.15, *p* < 0.0001) and also across seasons (V = 0.04, *p* < 0.0001) (Figure 3a,b). In 2023, locomotor issues were more frequent than expected under the null hypothesis (standardised residuals, std. res.: 12.7), while calving-related problems and recumbency became less common (std. res.: −3.5 and −10.6, respectively). Calving-related problems and recumbency were, however, more frequent than expected during winters (std. res.: 2.9 and 3.4, respectively). The frequency of the six most common subcategories of reasons for OFES varied significantly over the years as well (V = 0.09, *p* < 0.0001), with an increase in the frequency of traumas due to slips and falls (std. res. for 2023: 3.7) and a decrease in palsy and paralysis (std. res. for 2023: −6.0) (Figure 3c). These subcategories also varied in frequency across seasons (V = 0.04, *p* = 0.0011): the observed proportions of fractures and of hip dislocations and splits were larger than expected in spring (std. res.: 2.2 and 3.0, respectively), while acute traumas were more frequent in summer (std. res.: 2.7) and palsy and paralysis were common in winter (std. res.: 3.1) (Figure 3d).

**Figure 4:**
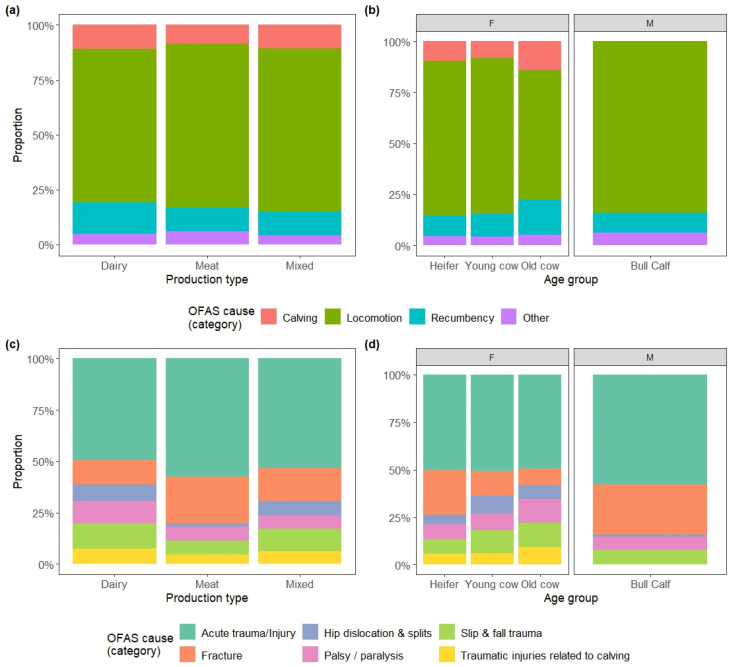
Distribution of the categories and subcategories of reasons for on-farm emergency slaughter (OFES) instances by farm production type and animal age group: (**a**) categories by production type; (**b**) categories by age group; (**c**) subcategories by production type; (**d**) subcategories by age group.

The reasons for OFES varied according to the type of production, in terms of frequency of both categories (Figure 4a, V = 0.04, *p* < 0.0001) and sub-categories (Figure 4c, V = 0.10, *p* < 0.0001). Locomotion problems were more frequently associated with meat and mixed production (std. res.: 2.6 and 3.3, respectively), while recumbency with dairy farms (std. res.: 4.6). Acute traumas and fractures were both more frequent in meat production (both std. res. > 3.8), whereas palsy and paralysis, hip dislocations and traumatic injuries related to calving were more common in dairy production (all std. res. > 2.8).

Differences in category (Figure 4b, V = 0.10, *p* < 0.0001) and sub-category (Figure 4d, V = 0.12, *p* < 0.0001) proportions were also found between the analysed female age groups (heifers, young cows, old cows). Calving-related problems and recumbency were more frequent than expected in old cows (both std. res. > 9.2), and locomotion problems were more common in heifers and young cows (std. res.: 5.0 and 11.4, respectively). Indeed, traumatic injuries related to calving as well as palsy and paralysis were more frequent in old cows (both std. res. > 6.1), while fractures were more common in heifers (std. res.: 12.8).

**Figure 5:**
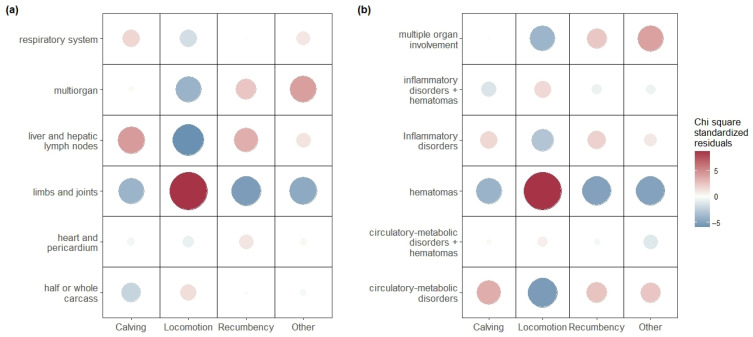
Associations between categories of reasons for on-farm emergency slaughter (OFES) and the outcome of post-mortem inspection in terms of exclusion reasons (**a**) and localisation (**b**). The size and colour of the dots represent the difference between observed and expected frequencies, indicating the contribution of each pair to the significant chi square tests.

Finally, significant differences were found among the OFES reasons (categories) and the outcome of post-mortem inspections, both in terms of condemnation sites (Figure 5a, V = 0.13, *p* = 0.0005) and the reasons for condemnation (Figure 5b, V = 0.12, *p* = 0.0005). The locomotion category was strongly associated with lesions at the limbs and joints (std. res.: 8.7) and particularly hematomas (std. res.: 8.9), while calving and recumbency were strongly associated with lesions located in the liver or hepatic lymph nodes (std. res. 4.3 and 3.4, respectively) and particularly with circulatory–metabolic disorders (std. res. 3.6 and 2.6, respectively).

## 4. Discussion

In this study, we examined data from over 12,000 cattle undergoing an OFES in almost 2000 Northern Italian farms between 2021 and 2023, detecting a downward temporal trend and significant differences in their occurrence based on farm and animal characteristics.

Locomotor disorders, particularly acute trauma and fractures, were the predominant reason for OFES, accounting for approximately 70% of all cases. This finding is consistent with previous studies in Czech Republic [23] and Ireland [24], where a similar prevalence (77%) was found. Although less frequent, locomotor problems were also reported as the main reason for OFES in a Norwegian [20] and a Canadian study [10] accounting for half or more of all cases. Acute traumas and injuries were the most common reasons of locomotor disorders, representing 40% of all OFES, which is consistent with the eligibility criteria for this emergency procedure. Trauma due to slips, falls and hip dislocations, together accounting for 15% of cases, were usually attributed to on-farm accidents involving machinery, structures, slippery floors or mounting behaviours. However, distinguishing injuries in otherwise healthy animals from those linked to underlying conditions, such as calving complications, remains challenging [10,35]. To reduce the incidence of these traumatic events, improvements in farm management [36] and timely animal care [15] should be adopted. Similarly to previous findings [24], fractures were common, though their prevalence varied by production type, being almost three times more frequent in beef farms than in dairy farms (17% versus 9%). This finding can be explained, at least partly, by the selection of cattle crossbreeds for rapid muscle growth at the expense of bone structure, with reduced bone vascular support [37]. In addition, the genetic characteristics of cattle can influence the reaction to handling: breeds selected for beef production seem to have a smaller flight zone size compared to dairy breeds [38,39]. Lameness accounted for less than 2% of OFES, which is in contrast with previous studies from Norway (21%) [20] and Canada, British Columbia (9%) [10]; this difference could be explained by different legislative frameworks and national or local guidelines. For instance, newly lame animals are considered suitable for OFES. The Norwegian national guidelines state that low-grade lame animals can be treated for up to seven days and then, if the treatment fails, OFES is permitted [9]. In contrast, the British Columbia guidelines state that OFES should not be allowed for chronic conditions, including lameness [10]. Locomotor disorders were strongly associated with post-mortem condemnation of limbs and joints, as well as to the presence of haematomas. These lesions reduce carcass value, limiting reducing the economic benefits of OFES. Limbs and joints were the most common exclusion localisation (36%). This is consistent with the OFES eligibility criteria, which include animals with limb injuries rendering them unfit for transport to a slaughterhouse (Regulation EC 1/2005). A recent Italian study on post-mortem inspection in dairy cows found a higher prevalence of muscle lesions in cattle undergoing an OFES compared to those slaughtered conventionally [40]. In particular, the presence of carcass haematomas may reflect poor handling and inadequate farm management [41]. Haematomas may not be apparent at ante-mortem inspection due to skin thickness, whereas post-mortem examination allows a direct assessment [42]. Considering the predominant role of locomotory disorders in OFES and their negative impact on carcass quality, improved preventive measures, such as early detection strategies and proper culling protocols [43], should be implemented to optimise this procedure.

Although less common than locomotor problems, recumbency accounted for a significant proportion (13%) of OFES. In particular, ‘palsy/paralysis’ was the most common reason for recumbency, especially in older cows. These results are similar to those reported in a previous study [20], but almost twice as high as those reported in another [10]. These discrepancies could be explained by different housing conditions, but also by some differences in the classification of OFES reasons, which hinders comparisons between studies. Our results show that most of the cases of OFES recorded as recumbency on veterinary certificates were due to non-traumatic reasons, which is consistent with the sample including mostly dairy cows. Indeed, recumbency in dairy cows is often associated with the onset of lactation as a consequence of periparturient hypocalcaemia or calving-related complications [36]. The prevention of such issues requires balanced nutrition, proper environmental management and timely interventions to reduce complications, especially in cases where the secondary damage (i.e., vascular and nerve damage) has a major impact on the prognosis [35,44].

Calving-related complications were also relatively common, being the reason for about 10% of all OFES. Their frequency was similar in dairy (11%), mixed (11%) and beef (9%) farms. This is in contrast with a previous study, which found three times more reproductive disorders in the beef sector than in the dairy sector [20]. These differences can be explained by the small number of female cattle from beef farms included in our study, with beef cows accounting for less than 1% of all OFES. Nevertheless, the representativeness of the sample should have remained mostly unaffected, since the distribution of OFES by sex and age group in our sample was similar to that in Italy during the same years [45]. In addition, calving-related problems may be generally under-represented as reasons of OFES, as a percentage of recumbency may be a consequence of calving [46]. Calving-related complications were positively associated with the condemnation of liver and hepatic lymph nodes, as well as the presence of circulatory-metabolic disorders. This result could be explained again by the predominance of dairy cows in the sample, in which hepatic steatosis is a frequent metabolic disorder [47,48]. In order to rapidly supply the incoming milk yield, the liver requires a large amount of metabolizable energy supplied by non-esterified fatty acids, which can ultimately lead to circulatory and metabolic disorders (e.g., hepatic steatosis) [49]. Even in its mild form, hepatic steatosis is associated with poor health and reduced reproductive performance [47]. The liver and its lymph nodes were the second most common exclusion localisation (21%) and were indeed associated with circulatory-metabolic disorders in 70% of cases (Table S4). To reduce the impact of calving-related complications and their consequences, it is essential to provide a balanced diet with the necessary nutrients, especially during the peripartum period [50], to maintain a clean environment and to ensure a prompt treatment for postpartum metabolic disorders [48].

Although the category ‘other’ also covered the less common reasons of OFES, in most cases the diagnoses within this category were vague (generic trauma) or improper (missing or illogical). Apart from data quality issues, this result may reflect some difficulties in using only external observation to accurately identify conditions that meet the eligibility criteria for OFES. Nonetheless, the positive association between the category ‘other’ and multiorgan exclusions suggests the presence of severe pathological conditions that could be compatible with an OFES.

The respiratory system was the third most frequent exclusion location, accounting for 14% of all cases. Similarly to other studies [40], the majority of respiratory lesions were of inflammatory nature (Table S4). Apart from a slight positive association with calving complications, these exclusions were evenly distributed across the main categories of OFES reasons, suggesting that these were secondary forms with no particular relationship to such categories. Indeed, those lesions seem to be more common in regularly slaughtered cattle, with previous studies reporting a prevalence more than twice as high [51,52].

The significant downward trend in OFES, with a 30% reduction between 2021 and 2023, could be explained by the refinement of the eligibility criteria for such procedures in Italy. In 2021, an audit by the European Commission’s Directorate-General for Health and Food Safety (DG SANTE) revealed a historical misclassification of cattle with metabolic disorders as eligible candidates. Following the audit, the Italian Ministry of Health issued a more refined interpretation of ‘accident’, emphasising that an animal must be free of symptoms attributable to infectious diseases, metabolic syndromes, or neurological disorders (Italian Ministry of Health Circular No. 13895/2022). Such a change has probably contributed to a stricter selection, thereby reducing the number of procedures performed. This is further supported by the decreasing trend in recumbency (−71%) and calving-related complications (−54%), while locomotor disorders have remained stable over the years. This result highlights the impact that evolving legislative frameworks can have on emergency slaughter protocols and approaches. Although a seasonal trend has been previously reported [40], in our study the effect of the season was very limited (V = 0.05), with a tendency for OFES to increase during the summer. High temperature and humidity have been reported as risk factors for mortality in dairy cows [53,54]. These environmental conditions can induce heat stress, which may result in tachypnoea, tachycardia, dehydration and, in severe cases, death [55]. However, this risk can vary depending on the rearing system and may be mitigated through grazing [56].

The occurrence of OFES cases varied also depending on the day of the week, with the highest frequency on Mondays and the lowest on Saturdays. Since slaughterhouses do not usually operate on the weekend, logistical constraints may lead to a delay in the OFES, with a negative impact on animal welfare. Furthermore, factors related to weather conditions or specific management practices could influence the timing of OFES procedures [22]. Mobile abattoirs units could be used to minimise the impact on animal welfare, providing an immediate resolution and avoiding the transport of unfit animals [57]. Another important disadvantage of OFES during the weekend is the increased cost of ante-mortem inspections, as an official veterinarian has to work overtime to carry out the inspection. A recent study suggested to address this issue by using online ante-mortem inspections, but this approach does not comply with current EU legislations [58].

Despite the relevant amount of data collected and analysed for this study, some important limitations must be acknowledged. Only 30% of condemnation data were available and, in many cases, it was not specified whether the exclusion was partial or total. Additionally, ante-mortem and post-mortem inspection data were obtained from hand-written certificates on free-text paper, 2–3% of which were incomplete or illogical, and the validity of such certificates has been questioned in previous studies [20]. Finally, digitising paper sources and categorising OFES reasons in a standardised way could have led to data entry errors. These issues highlight the need for electronic and standardised systems to facilitate data collection and improve data quality.

## 5. Conclusions

This study provides novel insights into on-farm emergency slaughter (OFES) in Italy, emphasising the importance of preventive management and prompt intervention in mitigating issues such as trauma-related locomotor disorders, which may impact both animal welfare and carcass quality.

The overall decline in OFES procedures observed during the study period is likely due to the implementation of stricter eligibility criteria. This underlines how regulatory refinement can influence veterinary decision-making and reduce inappropriate or borderline cases, emphasising the importance of clear, harmonised policy frameworks. Furthermore, to address logistical challenges and improve animal welfare outcomes, policymakers should consider supporting the use of mobile slaughter units, strengthening veterinary training and implementing electronic data collection systems to enhance traceability and inspection reliability.

While the analysed dataset was extensive, limitations such as inconsistent record-keeping (mostly due to handwritten forms) and non-standard terminology should be overlooked, suggesting a transition to an electronic system that only encompasses standardised data collection. Future research should prioritise harmonised data protocols and multi-country comparisons to improve our understanding of OFES practices. Continued monitoring is also required to evaluate the long-term effects of policy changes and support the development of sustainable emergency slaughter procedures that prioritise animal welfare.

## Figures and Tables

**Table 1:** Division into age categories and corresponding age ranges (in days) of the cattle included in the study.

Sex	Age Group	Age Range (Days)
Female	Female calves	Birth to 180
	Heifers	181–760 ^1^
	Young cows	761–1460
	Old cows	Over 1460
Male	Male calves	Birth to 180
	Bull calves	181–760
	Young bulls	761–1460
	Old bulls	Over 1460

^1^ Estimated age at first calving.

**Table 2:** List of categories and sub-categories of on-farm emergency slaughter (OFES) reasons in cattle, including their characterisation criteria (modified from [20,24]).

Category	Subcategory	Criteria *
Locomotion	Fracture	Fracture or suspected fracture (foreleg, hindleg, leg—not specified, spinal/back, hip/pelvic; shoulder/neck, not specified)
	Lameness	Lameness (foreleg, hindleg, lame—not specified)
	Acute Trauma/Injury	Generic trauma or injury to the legs, including acute soft tissue trauma (foreleg, hindleg, leg—not specified, spinal/back, hip/pelvic, shoulder/neck, not specified)
	Hip dislocation and splits	Animals with hip dislocation or splits
	Mounting trauma	Mounting (no information on the type of injury)
	Slip and fall trauma	Slips or falls within the barn (no information on the type of injury)
	Abscess or phlegmon	Abscess or phlegmon to legs
	Other in locomotion	Leg malformation or defects due to other reasons
Recumbency	Unable to stand	Unable to stand or walk for unknown reason
	Palsy and paralysis	Downer cows (using ‘palsy’ or ‘paralysis’ words) for unknown reason and involving also ‘hindquarters palsy’
	Metabolic syndrome	Metabolic disorders or collapse for unknown reason
Calving-related issues	Stillbirth	Stillbirth or abortion
	Dystocia	Dystocia or calving difficulties, including uterine torsion
	Uterine and obstetrics affections	Cases with a current vaginal or uterine prolapse, including also uterine lacerations or haemorrhages
	Traumatic injuries related to calving	Neuromuscular damage due to compression from trauma occurring during calving, including dislocation of the pelvic bones
	Palsy and paralysis after calving	Downer cows (using ‘palsy’ or ‘paralysis’ words) but reported after calving
	Metabolic syndrome after calving	Metabolic disorders or collapse but reported after calving
Other	Generic trauma	Trauma (no information on reasons and localisation)
	Aggressiveness	Animals that cannot be caught or loaded on truck, aggressive and uncontrollable.
	Cardiac	Cardiovascular disorders
	Digestive	Digestive disorders (ruminal bloat, displaced abomasum, intestinal torsion)
	Eye	Eye disorders, including blindness
	Foreign body syndrome	Traumatic reticuloperitonitis
	Head	Head injuries, including the horn
	Integument	Skin wounds but unknown body localization
	Mastitis	Mastitis
	Respiratory	Pneumonia or dyspnoea
	Udder damage	Trauma or wounds to the udder
	Empty or illogical	No reason provided or data entry errors
	Other	Other (e.g., post-operative complications without additional information)

* Based on the reasons reported in veterinary certificates.

**Table 3:** List of reasons for condemnation, observed during post-mortem inspections of cattle subject to on-farm emergency slaughter (OFES), including their characterisation criteria.

Reasons for Condemnation	Criteria ^1^
Circulatory and metabolic disorders	Jaundice, pressure ulcers, hypostases, myxomatous mitral valve diseases, cardiac exhaustion, nephrosis, steatosis, telangiectasia
Contamination	Faecal or food contamination
Fracture	Open or closed fracture
Haematoma	Tissue injury with rupture of the vascular supply and accumulation of blood and serum
Inflammatory disorders	Abscesses, phlegmons, necrosis, hepatitis, hepatic adhesions, pericarditis, endocarditis, peritonitis, reticulitis, enteritis, arthritis, pneumonias, bronchitis, tracheitis, pleuritis, emphysema, renal cysts, splenitis, nephritis
Meat organoleptic changes	Noticeable changes in the colour and consistency of the muscle mass on visual inspection
Multiorgan impairment	Multiple organs excluded due to being affected by one or more diseases
Parasitic lesions	Lesions attributable to the presence or migration of parasites
Wounds	Presence of an open wound
Generic trauma	Type of traumatic lesion not described

^1^ Based on the reasons reported in veterinary certificates.

**Table 4:** Distribution of 12,052 on-farm emergency slaughter (OFES) instances, carried out on 1858 cattle farms in Northern Italy, grouped by production system, sex or animal category.

Group	Sub-Group	2021 OFES No. (%)	2022 OFES No. (%)	2023 OFES No. (%)	Total OFES No. (%)
Production system	Dairy	3821 (78.8)	3007 (78.3)	2686 (79.8)	9514 (78.9)
	Mixed	643 (13.3)	469 (12.2)	385 (11.4)	1497 (12.4)
	Beef	383 (7.9)	362 (9.4)	296 (8.8)	1041 (8.6)
Sex	Female	4581 (94.5)	3588 (93.5)	3190 (94.7)	11359 (94.2)
	Male	266 (5.5)	250 (6.5)	177 (5.3)	693 (5.8)
Animal category	Female calf	10 (0.2)	0 (0)	4 (0.1)	14 (0.1)
	Heifer	557 (11.5)	500 (13)	442 (13.1)	1499 (12.4)
	Young cow	1787 (36.9)	1451 (37.8)	1324 (39.3)	4562 (37.9)
	Old cow	2227 (45.9)	1637 (42.7)	1420 (42.2)	5284 (43.8)
	Male calf	3 (0.1)	4 (0.1)	3 (0.1)	10 (0.1)
	Bull calf	244 (5.0)	229 (6.0)	168 (5.0)	641 (5.3)
	Young bull	18 (0.4)	14 (0.4)	6 (0.2)	38 (0.3)
	Old bull	1 (<0.1)	3 (0.1)	0 (0)	4 (<0.1)

**Table 5:** Distribution of 12,052 on-farm emergency slaughter (OFES) instances, carried out in 2021–2023 on 1858 cattle farms in Northern Italy, grouped by reason (category and sub-category).

Reason (Category)	Reason (Sub-Category)	OFES in Dairy Farms No. (%)	OFES in Mixed Farms No. (%)	OFES in Beef Farms No. (%)	OFES in All Farms No. (%)
Locomotion	Fracture	890 (9.4)	188 (12.6)	173 (16.6)	1251 (10.4)
	Lameness	149 (1.6)	27 (1.8)	41 (3.9)	217 (1.8)
	Acute trauma/injury	3703 (38.9)	629 (42.0)	440 (42.3)	4772 (39.6)
	Hip dislocation and splits	607 (6.4)	83 (5.5)	12 (1.2)	702 (5.8)
	Mounting trauma	232 (2.4)	35 (2.3)	32 (3.1)	299 (2.5)
	Slip and fall trauma	912 (9.6)	129 (8.6)	49 (4.7)	1090 (9.0)
	Abscess or phlegmon	24 (0.3)	2 (0.1)	1 (0.1)	27 (0.2)
	Other in locomotion	4 (<0.1)	0 (0)	0 (0)	4 (<0.1)
Recumbency	Unable to stand	396 (4.2)	59 (3.9)	52 (5.0)	507 (4.2)
	Palsy/paralysis	825 (8.7)	76 (5.1)	52 (5.0)	953 (7.9)
	Metabolic syndrome	112 (1.2)	21 (1.4)	4 (0.4)	137 (1.1)
Calving-related problems	Stillbirth	7 (0.1)	0 (0)	0 (0)	7 (0.1)
	Dystocia	107 (1.1)	25 (1.7)	30 (2.9)	162 (1.3)
	Uterine and obstetrics	38 (0.4)	8 (0.5)	12 (1.2)	58 (0.5)
	Traumatic injuries	550 (5.8)	72 (4.8)	33 (3.2)	655 (5.4)
	Palsy/paralysis	164 (1.7)	35 (2.3)	7 (0.7)	206 (1.7)
	Metabolic syndrome	147 (1.5)	15 (1.0)	2 (0.2)	164 (1.4)
Other	Generic trauma	280 (2.9)	31 (2.1)	32 (3.1)	343 (2.8)
	Aggressiveness	1 (<0.1)	2 (0.1)	4 (0.4)	7 (0.1)
	Cardiac	7 (0.1)	1 (0.1)	1 (0.1)	9 (0.1)
	Digestive	79 (0.8)	5 (0.3)	6 (0.6)	90 (0.7)
	Eye	2 (<0.1)	1 (0.1)	1 (0.1)	4 (0.0)
	Foreign body syndrome	47 (0.5)	10 (0.7)	8 (0.8)	65 (0.5)
	Head	8 (0.1)	6 (0.4)	5 (0.5)	19 (0.2)
	Integument	4 (<0.1)	1 (0.1)	0 (0)	5 (<0.1)
	Mastitis	13 (0.1)	0 (0)	0 (0)	13 (0.1)
	Respiratory	9 (0.1)	3 (0.2)	8 (0.8)	20 (0.2)
	Udder damage	9 (0.1)	1 (0.1)	0 (0)	10 (0.1)
	Empty or illogical	176 (1.8)	30 (2.0)	33 (3.2)	239 (2.0)
	Other	12 (0.1)	2 (0.1)	3 (0.3)	17 (0.1)

**Table 6:** Distribution of carcass and organ condemnation in 3181 on-farm emergency slaughter (OFES) instances carried out in 2021–2023 on 1061 cattle farms in Northern Italy.

Condemnation Sites	Cases No. (%)
Limbs and joints	1139 (35.8)
Liver and hepatic lymph nodes	676 (21.3)
Respiratory system	454 (14.3)
Multiorgan	394 (12.4)
Half or whole carcass	245 (7.7)
Heart and pericardium	97 (3.0)
Empty or illogical	79 (2.5)
Spleen and kidneys	44 (1.4)
Gastrointestinal system and peritoneum	31 (1.0)
Head and oral cavity	16 (0.5)
Integumentary system and mammal gland	6 (0.2)

## Data Availability

The data presented in this study are available upon reasonable request from the corresponding author, provided that the names of the slaughterhouses and farms are anonymised, due to the sensitive nature of such data.

## Supplementary Materials

The following supporting information can be downloaded at: https://www.mdpi.com/article/10.3390/ani15152239/s1. Table S1: OFES certificate; Table S2: Distribution of OFES instances by sex and animal category for each production system; Table S3: Distribution of exclusions for locomotion category; Table S4: Distribution of carcass and organ exclusions.

## Abbreviations

The following abbreviations are used in this manuscript:

OFES	On-farm emergency slaughter
LCVA	Local Competent Veterinary Authority
