# Supplementary material for: Occurrence and Reasons for On-Farm Emergency Slaughter (OFES) in Northern Italian Cattle
Source: Animals (Basel). 2025 Jul 30;15(15):2239. doi: 10.3390/ani15152239 (PMC12345588; doi:10.3390/ani15152239)
Supplement: Supplementary file 1 [file animals-15-02239-s001.zip › Table_S1_Fusi_et_Al_OFES.pdf]

Occurrence and Reasons for On-Farm Emergency Slaughter (OFES) in Northern Italian Cattle

Francesca Fusi, Camilla Allegri, Alessandra Gregori, Claudio Monaci, Sara Gabriele, Tiziano Bernardo, Valentina Lorenzi, Claudia Romeo, Federico Scali, Lucia Scuri, Giorgio Bontempi, Maria Nobile, Luigi Bertocchi, Giovanni Loris Alborali, Adriana Ianieri and Sergio Ghidini

Table S1. Format of OFES certificate, which is completed by the Official Veterinarian, in accordance with the Commission Implementing Regulation (EU) 2020/2235 and the Italian Ministry of Health Circular No. 13895/2022.

MODEL 1

HEALTH CERTIFICATE
in case of emergency slaughter outside the slaughterhouse

Official veterinarian's name:

Registration number with the Register of Veterinary Surgeons and associated Province:
____

1. Animal identification:
Species:

Number of animals:

Identification mark:

Owner/keeper of the animals:

2. Place of emergency slaughter
Address:

BDN code and any identification of the housing box:

3. Destination of slaughtered animals for the purpose of slaughter
the animals will be transported to the following slaughterhouse:
____ using the following means of transport:

4. Other relevant information:

5. Declaration
I the undersigned declare that:

- the animals above were examined before slaughter at the aforementioned farm at _______ (time hh:mm) on ________ (date) and were deemed fit for slaughter,
- they were slaughtered at ________ (time hh:mm) on ________ (date) and the slaughter and bleeding were carried out correctly by trained personnel (Reg. 1099/2009),
- the reason for the emergency slaughter was:

- the following observations were made regarding the health and welfare of the animals: ________________________
- the animals were administered the following drug treatments in the last 90 days: _______________
- the records and documentation relating to these animals comply with current regulations and did not determine the prohibition of slaughtering them.

Done at (place) ____ on (date dd/mm/aa) _______________

Stamp

(Veterinarian Public Officer's signature)

MODEL 2

SPECIMEN OF REPORT/SCU OF UNFAVORABLE ANTE MORTEM INSPECTION IN CASE OF REQUEST FOR EMERGENCY SLAUGHTER OUTSIDE THE SLAUGHTERHOUSE

Official Veterinarian's Name:
Veterinary Surgeons Register Number:
____

1. **Animal Identification**

Species:

Number of Heads:

Identification Mark:

2. **Place of Emergency Slaughter**

Address:

BDN Code and Possible Identification of the Stabling Box*:

3. **Other Relevant Information**
4. **Declaration**

The undersigned declares that:

- the animals mentioned above were examined before slaughter at the farm indicated above at ________. (time hh:mm) on _________(date) and deemed NOT suitable for slaughter,
- the reason for the refusal of emergency slaughter was: ____________________________
- observations on the health and welfare of the animals: ____________________________
- measures taken: ____.
- the animals were administered the following drug treatments in the last 90 days:

Done at (place)__________________________________on (date dd/mm/yy) _________________________

Stamp

(Official Veterinarian's Signature)

*Optional

This certificate must be issued in at least two copies: 1 for the completing veterinary surgeon and 1 to be kept in the farm's records.
