# Supplementary material for: Occurrence and Reasons for On-Farm Emergency Slaughter (OFES) in Northern Italian Cattle
Source: Animals (Basel). 2025 Jul 30;15(15):2239. doi: 10.3390/ani15152239 (PMC12345588; doi:10.3390/ani15152239)
Supplement: Supplementary file 1 [file animals-15-02239-s001.zip › Table_S2_Fusi_et_Al_OFES.pdf]

Occurrence and Reasons for On-Farm Emergency Slaughter (OFES) in Northern Italian Cattle

Francesca Fusi, Camilla Allegri, Alessandra Gregori, Claudio Monaci, Sara Gabriele, Tiziano Bernardo, Valentina Lorenzi, Claudia Romeo, Federico Scali, Lucia Scuri, Giorgio Bontempi, Maria Nobile, Luigi Bertocchi, Giovanni Loris Alborali, Adriana Ianieri and Sergio Ghidini

Table S2. Distribution of 12,052 on-farm emergency slaughter (OFES), carried out on 1,858 cattle farms in Northern Italy, grouped by sex and animal category for each production system and total OFES.

Group	Subgroup	OFESs * in Dairy Farms No. (%)	OFESs * in Mixed Farms No. (%)	OFESs * in Meat Farms No. (%)	OFESs * in All Farms No. (%)
Sex	Female	9467 (83.3)	1396 (12.3)	496 (4.4)	11359 (100)
	Male	47 (6.8)	101 (14.6)	545 (78.6)	693 (100)
Animal category	Female calf	9 (64.3)	3 (21.4)	2 (14.3)	14 (100)
	Heifer	868 (57.9)	247 (16.5)	384 (25.6)	1499 (100)
	Young cow	3976 (87.2)	533 (11.7)	53 (1.2)	4562 (100)
	Old cow	4614 (87.3)	613 (11.6)	57 (1.1)	5284 (100)
	Male calf	0 (0)	0 (0)	10 (100)	10 (100)
	Bull calf	34 (5.3)	98 (15.3)	509 (79.4)	641 (100)
	Young bull	10 (26.3)	3 (7.9)	25 (65.8)	38 (100)
	Old bull	3 (75.0)	0 (0)	1 (25.0)	4 (100)

* On farm emergency slaughters.
