# Supplementary material for: Occurrence and Reasons for On-Farm Emergency Slaughter (OFES) in Northern Italian Cattle
Source: Animals (Basel). 2025 Jul 30;15(15):2239. doi: 10.3390/ani15152239 (PMC12345588; doi:10.3390/ani15152239)
Supplement: Supplementary file 1 [file animals-15-02239-s001.zip › Table_S3_Fusi_et_Al_OFES.pdf]

Occurrence and Reasons for On-Farm Emergency Slaughter (OFES) in Northern Italian Cattle

Francesca Fusi, Camilla Allegri, Alessandra Gregori, Claudio Monaci, Sara Gabriele, Tiziano Bernardo, Valentina Lorenzi, Claudia Romeo, Federico Scali, Lucia Scuri, Giorgio Bontempi, Maria Nobile, Luigi Bertocchi, Giovanni Loris Alborali, Adriana Ianieri and Sergio Ghidini

Table S3. Distribution of exclusions with body localisations and type of injuries, within the locomotion category.

Body Localisation	Fracture (%)	Lameness (%)	Acute Trauma or Injury (%)	Total (%)
Foreleg	140 (11.2)	22 (10.1)	282 (5.9)	444 (7.1)
Hindleg	880 (70.3)	89 (41)	3311 (69.4)	4280 (68.6)
Leg (not specified)	31 (2.5)	0 (0)	502 (10.5)	533 (8.5)
Spinal or back	10 (0.8)	0 (0)	351 (7.4)	361 (5.8)
Hip or pelvic	147 (11.8)	0 (0)	288 (6)	435 (7)
Shoulder or neck	2 (0.2)	0 (0)	25 (0.5)	27 (0.4)
Not specified	41 (3.3)	106 (48.8)	13 (0.3)	160 (2.6)
Total	1251	217	4772	6240
