# Supplementary material for: Occurrence and Reasons for On-Farm Emergency Slaughter (OFES) in Northern Italian Cattle
Source: Animals (Basel). 2025 Jul 30;15(15):2239. doi: 10.3390/ani15152239 (PMC12345588; doi:10.3390/ani15152239)
Supplement: Supplementary file 1 [file animals-15-02239-s001.zip › Table_S4_Fusi_et_Al_OFES.pdf]

Occurrence and Reasons for On-Farm Emergency Slaughter (OFES) in Northern Italian Cattle

Francesca Fusi, Camilla Allegri, Alessandra Gregori, Claudio Monaci, Sara Gabriele, Tiziano Bernardo, Valentina Lorenzi, Claudia Romeo, Federico Scali, Lucia Scuri, Giorgio Bontempi, Maria Nobile, Luigi Bertocchi, Giovanni Loris Alborali, Adriana Ianieri and Sergio Ghidini

Table S4. Distribution of carcass and organ exclusions, in 3,181 on-farm emergency slaughters carried out in 2021–2023 on 1,061 cattle farms in Northern Italy, for each localisation.

Exclusion Localisations	Condemnation Cause	Cases No. (%)
Empty or illogical	Empty or illogical	79 (2.5)
Gastrointestinal system and peritoneum	Inflammatory disorders	30 (0.9)
	Inflammatory disorders + hematomas	1 (<0.1)
Half or whole carcass	Circulatory-metabolic disorders	50 (1.6)
	Circulatory-metabolic disorders + hematomas	3 (0.1)
	Contamination	1 (<0.1)
	Generic trauma	6 (0.2)
	Hematomas	103 (3.2)
	Inflammatory disorders	33 (1.0)
	Inflammatory disorders + hematomas	2 (0.1)
	Meat organoleptic changes	33 (1.0)
	Meat organoleptic changes + hematomas	14 (0.4)
Head and oral cavity	Contamination	6 (0.2)
	Hematomas	3 (0.1)
	Inflammatory disorders	6 (0.2)
	Inflammatory disorders + hematomas	1 (<0.1)
Heart and pericardium	Circulatory-metabolic disorders	4 (0.1)
	Inflammatory disorders	85 (2.7)
	Inflammatory disorders + hematomas	7 (0.2)
	Parasitic lesions	1 (<0.1)
Integumentary system and mammal gland	Hematomas	1 (<0.1)
	Inflammatory disorders	4 (0.1)
	Inflammatory disorders + hematomas	1 (<0.1)
Limbs and joints	Fracture	18 (0.6)
	Generic trauma	30 (0.9)
	Hematomas	993 (31.2)
	Inflammatory disorders	63 (2.0)
	Wounds	35 (1.1)
Liver and hepatic lymph nodes	Circulatory-metabolic disorders	463 (14.6)
	Circulatory-metabolic disorders + hematomas	60 (1.9)
	Contamination	1 (<0.1)
	Inflammatory disorders	122 (3.8)
	Inflammatory disorders + hematomas	16 (0.5)
	Parasitic lesions	13 (0.4)
	Parasitic lesions + hematomas	1 (<0.1)
Multiorgan	Multiple organ involvement	343 (10.8)
	Multiple organ involvement + hematomas	51 (1.6)
Respiratory system	Circulatory-metabolic disorders	9 (0.3)
	Contamination	3 (0.1)
	Inflammatory disorders	379 (11.9)
	Inflammatory disorders + hematomas	63 (2.0)
Spleen and kidneys	Circulatory-metabolic disorders	12 (0.4)
	Inflammatory disorders	25 (0.8)
	Inflammatory disorders + hematomas	7 (0.2)
